# Antipsychotic monitoring in dementia: quality of completion of antipsychotic monitoring forms in an older adult mental health service

**DOI:** 10.1192/bjb.2021.70

**Published:** 2022-10

**Authors:** Helen Anderson, Anna Kolliakou, Daniel Harwood, Nicola Funnell, Robert Stewart, Delia Bishara

**Affiliations:** 1 South London and Maudsley NHS Foundation Trust, London, UK; 2 Institute of Psychiatry, Psychology and Neuroscience, London, UK

**Keywords:** Antipsychotics, dementia, monitoring, forms, prescribing

## Abstract

**Aims and method:**

To support safe prescribing of antipsychotics in dementia, antipsychotic monitoring forms were embedded into our electronic health records. We present a review of the data collected on these forms to assess prescribing and identify areas for improvement in our practice and processes. Data were extracted from the structured fields of antipsychotic initiation and review forms completed between 1 January 2018 and 31 January 2020.

**Results:**

We identified gaps in practice where improvements could be made, mainly with regard to physical health monitoring (and particularly electrocardiograms, performed in only 50% of patients) and the low (less than 50%) recorded use of non-pharmacological interventions for behavioural and psychological symptoms of dementia. In addition, antipsychotic treatment was continued despite lack of benefit in almost 10% of reviews.

**Clinical implications:**

We advocate for recommendations on physical health monitoring of people with dementia taking antipsychotics to be added to the National Institute for Health and Care Excellence guidance on dementia and the Prescribing Observatory for Mental Health (POMH-UK) national audit.

The use of antipsychotic drugs in people with dementia is associated with an increased risk of cerebrovascular adverse events and greater mortality.^1–3^ Concerns over the years about their use to treat the behavioural and psychological symptoms of dementia (BPSD) have led to a national drive to reduce their inappropriate use and to improve the safety of prescribing and monitoring of antipsychotics in this population.

The Prescribing Observatory for Mental Health (POMH-UK) is a national initiative aiming to improve prescribing practices in psychiatry. The prevalence and quality of antipsychotic prescribing in dementia has been audited in 2011, 2012 and 2016, with standards derived from the National Institute for Health and Care Excellence (NICE) guidelines for dementia. The South London and Maudsley NHS Foundation Trust (SLaM) has participated in all relevant POMH-UK audits and is committed to improving prescribing practice in this area. In 2017 SLaM introduced electronic antipsychotic monitoring forms, which are embedded into electronic health records and include the prescribing and monitoring standards required when prescribing an antipsychotic in a patient with dementia, all of which are also included in the POMH-UK audit. The forms also allow for collection of physical health monitoring data, which is not required in the POMH-UK audit but is clinically important and is recommended in the Maudsley Prescribing Guidelines (14th edition).^4^ An ‘initiation form’ is completed at initiation of the antipsychotic in a patient with dementia and a ‘review form’ is completed every time the drug is reviewed for its efficacy and tolerability. It was hoped that the forms would act as tools to facilitate decision-making and encourage safe monitoring of antipsychotics in dementia, as well as facilitating data collection for audit purposes.

An earlier SLaM quality improvement project developed an algorithm to identify patients with dementia receiving an antipsychotic and checked that the antipsychotic monitoring forms had been completed. A mechanism was put in place to alert teams when the required initiation forms had not been completed. Following this project, monitoring form completion more than doubled (from 21.6% in October 2017 to 58.0% in January 2019).^5^ However, for the forms to fulfil their role, the quality of information recorded needs to be high as well as the form completion rate. Also, since the introduction of the forms, the rate of antipsychotic prescribing in those with dementia in SLaM has remained static. This throws into question whether clinicians are using the forms to determine whether the antipsychotic is necessary and appropriate, and indeed whether the forms help to rationalise prescribing in this area or whether they have become a ‘tick-box’ exercise.

The project described here aimed to review the information recorded on the antipsychotic monitoring forms. This was to identify whether the POMH-UK audit information is being captured, to better understand prescribing practices and identify areas for improvement in our practice and processes.

## Method

### Setting

Data were collected from the SLaM Biomedical Research Centre (BRC) Case Register, a database of anonymised clinical data from the electronic health records (EHRs) of patients receiving care from the trust.^6,7^ The register contains over 500 000 de-identified patient records, which are made available for research through the Clinical Record Interactive Search (CRIS) application.^8^ CRIS was developed at SLaM in 2008 and has been approved for secondary analysis (Oxford C Research Ethics Committee, reference 18/SC/0372). All research proposals using CRIS require approval by a patient-led oversight committee.

### Data source and extraction

We extracted data from the structured fields of our electronic antipsychotic initiation and review monitoring forms. Copies of the blank forms are available in the supplementary material available at https://doi.org/10.1192/bjb.2021.70. We collected all initiation and review forms completed between 1 January 2018 and 31 January 2020. We then discarded any forms for patients where an antipsychotic was being used for a primary psychotic illness. The remaining forms were thus focused on antipsychotic prescribing as treatment for BPSD.

We first reviewed key clinical information on the initiation forms, including dementia subtype and severity. We recorded the professional group of the person completing the form. We then assessed the content of the forms against the POMH-UK audit practice standards.

#### POMH-UK audit standard 1: the clinical indications for antipsychotic treatment should be clearly documented in the clinical records

On the initiation forms, respondents select the intended target symptoms, choosing one or more of the following: delusions, hallucinations, anxiety, depression, disturbed sleep, agitation, distress, verbal aggression, physical aggression, disinhibited behaviour, resisting help with personal care, wandering/restlessness, vocalisation and ‘other’.

#### POMH-UK audit standard 2: before prescribing antipsychotic medication for BPSD, likely factors that may generate, aggravate or improve such behaviours should be considered

Respondents are asked whether (reversible) generating/aggravating factors have been considered: depression, pain, anxiety, side-effects of other medications and physical illness (e.g. constipation, urinary tract infection, chest infection, heart failure). Our expectation was that all five of these common problems have been considered prior to starting an antipsychotic for BPSD. There is also an option to document ‘other causes’ considered.

The next section of the form asks respondents to record any non-pharmacological methods tried prior to considering antipsychotic treatment. There is a list of four suggested interventions: review of social and personal activities, changes in staff approach, changes to environment and watchful waiting/monitoring. There is an option for ‘other’ and a free-text box for comments.

#### POMH-UK audit standard 3: the potential risks and benefits of antipsychotic medication should be considered and documented by the clinical team, prior to initiation

This form asks whether a risk–benefit analysis has been performed, giving the options of ‘yes’, ‘no’ or ‘not applicable’. This field is mandatory: the form cannot be submitted without this information. There is then a free-text box in which the clinician is asked to add a comment.

#### POMH-UK audit standard 4: the potential risks and benefits of antipsychotic medication should be discussed with the patient and/or carer(s), prior to initiation

In the corresponding part of the form, the respondent must indicate ‘yes’, ‘no’ or ‘not applicable’ regarding whether there was a risk–benefit discussion with the patient, a relative or professional carer. There is also a free-text box to add comments about the risk–benefit discussion.

#### POMH-UK audit standard 5: medication should be regularly reviewed, and the outcome of the review should be documented in the clinical records.

This standard requires that the medication review should take account of (a) therapeutic response and (b) possible adverse effects.

On the review forms, respondents are asked to judge the antipsychotic effect on the target BPSD symptoms using one of the following options: ‘better’, ‘no change’, ‘not certain’ or ‘worse’. Completion of this field is also mandatory.

The review forms also require the recording of whether or not the patient has experienced one of the following adverse effects: sedation, falls, impaired mobility, chest infection, anticholinergic symptoms, rigidity, tremor, transient ischemic attack (TIA), cerebrovascular accident (CVA), low blood pressure. Respondents may also choose ‘other’. Again, this is obligatory and required for submission of the form. Respondents can choose whether or not to document any comments they may have on the specific side-effects.

The respondent must record the outcome of the review with regard to the antipsychotic drug. They are given the following options: ‘no change’, ‘stop antipsychotic’, ‘increase dose’, ‘reduce dose’ or ‘change antipsychotic’.

### Physical health monitoring

The Maudsley Prescribing Guidelines recommend that all patients with dementia prescribed antipsychotic drugs should have the following tests at baseline, 3 months and then annually: blood pressure and pulse, weight, electrocardiogram (ECG) and full blood count, urea and electrolytes, including estimated glomerular filtration rate, fasting glucose or glycated haemoglobin (HbA_1c_), lipids, liver function tests and prolactin.^4^ These recommendations are based on NICE guidance for people taking antipsychotics for the treatment of schizophrenia.

The initiation form requires respondents to state whether the patient has had each of these tests within the past 3 months. On the review forms, respondents are asked to record whether the physical health parameters have been recorded in the past year. This information is mandatory. There is also the option to add a comment.

### Data analysis

Results were generated from descriptive statistics. Microsoft Excel for Mac version 16.40 was used for all analyses. The forms contained free-text boxes to add extra information or comments. The authors reviewed these and identified themes which are included in the results.

## Results

There were 6673 patients identified who had received a primary or secondary ICD-10 diagnosis of dementia in SLaM at any point between 1 January 2018 and 31 January 2020. Over this time period, 249 antipsychotic initiation forms were completed. We included 203 initiation forms after excluding 46 forms that indicated the antipsychotic was being prescribed for primary psychotic illness. In total, 690 review forms were also completed in this period and we included 504 of these after excluding the ones where the antipsychotic was prescribed for primary psychotic illness.

### Initiation forms

Supplementary Table 1 outlines key clinical patient information collected on initiation forms. Doctors completed the highest proportion of forms (47.3%), followed by nurses (31.0%), occupational therapists (16.7%), psychologists (1.5%), administrators (1%) and ‘other’ (2.5%).

**Fig. 1:**
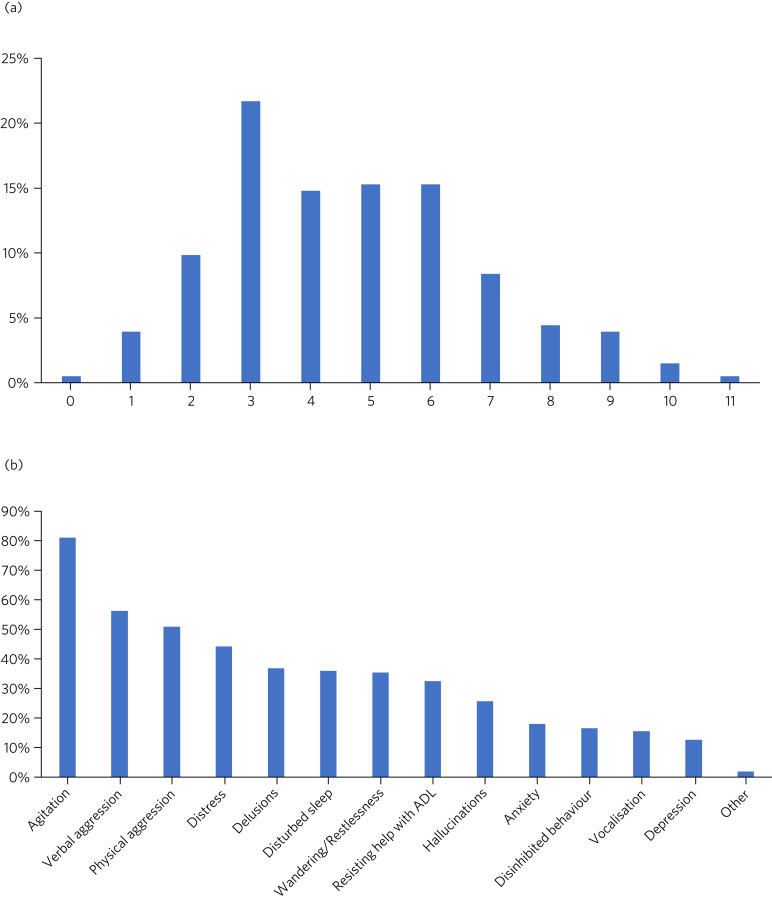
Target symptoms (data from initiation form). (a) Proportion of patients with the specified number of target symptoms for antipsychotic treatment (*n* = 203). (b) Proportion of patients with the specified symptom documented as a target for antipsychotic treatment (*n* = 203). ADL, activities of daily living.

Only one form had no target symptoms recorded. Figure 1(a) outlines the number of target symptoms selected per form. The median number of target symptoms selected was four. Agitation was the most common target symptom, followed by verbal and physical aggression (Fig. 1(b)).

All five of the generating/aggravating factors for BPSD had been considered in 68.4% of forms. Additionally, 50.0% indicated that ‘other’ causes had been considered. Ninety-eight (48.3%) of the forms recorded that non-pharmacological interventions were tried, either by selecting from the suggested interventions or by completing the free-text box. Review of the comments revealed that in many cases inventive and personalised approaches were used, often enlisting the help of the patient's family members and carers. A further theme from the comments section was that in some instances the patient was felt to be too unwell or the risks felt to be too great to withhold antipsychotic treatment while non-pharmacological methods were tried.

One hundred and eighty-nine forms (93.1%) recorded that a risk–benefit analysis had been performed. On six of the forms (3.0%) the clinician stated that a risk–benefit analysis was not performed, and on eight of the forms (3.9%) the clinician felt that the risk–benefit analysis was ‘not applicable’.

Discussion with the patient, relative or a carer was recorded in 182 cases (89.7%) and an additional comment was made on 121 of the forms (59.6%). Comments included documenting that the patient did not have the capacity to make the decision to take the antipsychotic and therefore a best interests decision was made (under the Mental Capacity Act 2005); or that a discussion took place with the patient or relative about the risks of shortened life expectancy and CVA. Documented risks of not prescribing the medication included carer stress, breakdown of placement, deteriorating self-care, risks to others, significant patient distress and decreased quality of life.

#### Baseline physical health monitoring

**Fig. 2:**
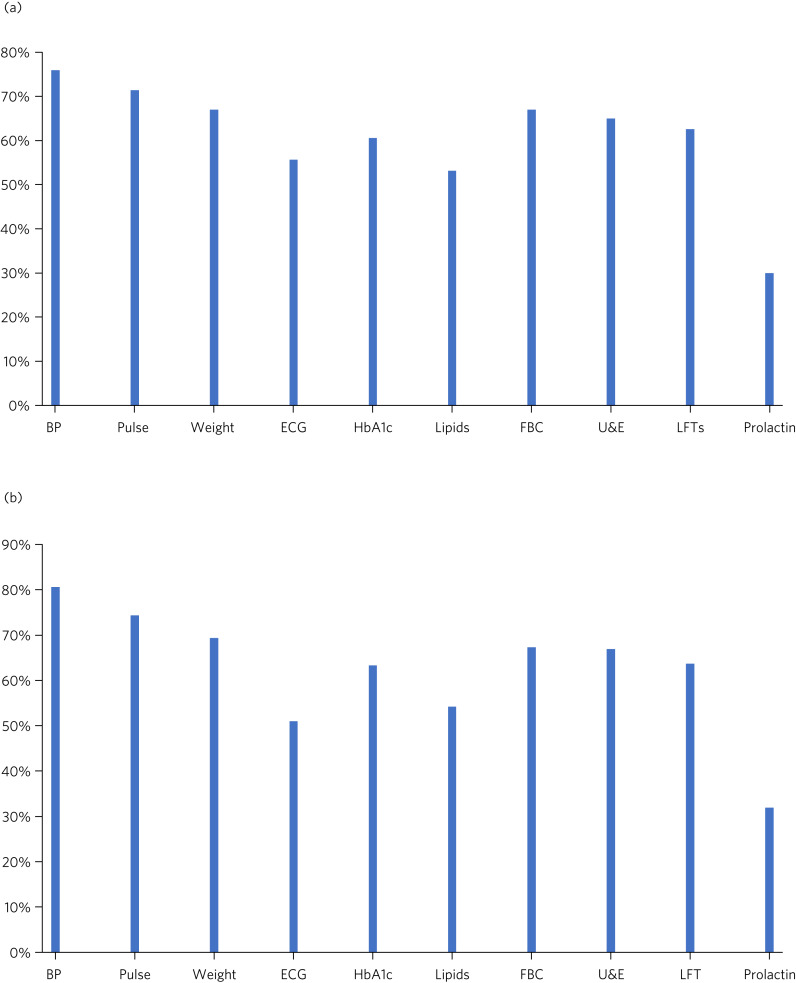
Physical health monitoring. (a) Proportion of patients who had the specified physical health parameter measured at baseline (*n* = 203) (data from initiation form). (b) Proportion of patients who had the specified physical health parameter measured on review (*n* = 504) (data from review form). BP, blood pressure; ECG, electrocardiogram; HbA_1c_, glycated haemoglobin; FBC, full blood count; U & E, urea and electrolytes; LFTs, liver function tests

Figure 2 illustrates the physical health parameters measured at baseline and at review of the antipsychotic drug. Figure 2(a) shows the proportion of patients who had each specified physical health parameter measured at baseline. Results varied: over 70% of patients had blood pressure and pulse monitoring, around 55% had ECG monitoring and only 30% had prolactin levels taken. A common response in the comments section was that the psychiatric team had asked the general practitioner (GP) to carry out the investigations and that the results were awaited. This comment was made on 24 of the forms (11.8%). In some cases, a comment had been made that the patient was too distressed to tolerate an ECG or blood tests (3.5%) or that the patient was unable to access an ECG because they were bed-/housebound (2.5%).

### Review forms

In the review forms, therapeutic response to the antipsychotic was described as follows: 55.6% of patients were considered ‘better’, there was ‘no change’ in 28.0%, in 10.1% of cases assessors were ‘not certain’ and in 6.3% it was felt that the BPSD symptoms were ‘worse’ on the antipsychotic drug.

**Fig. 3:**
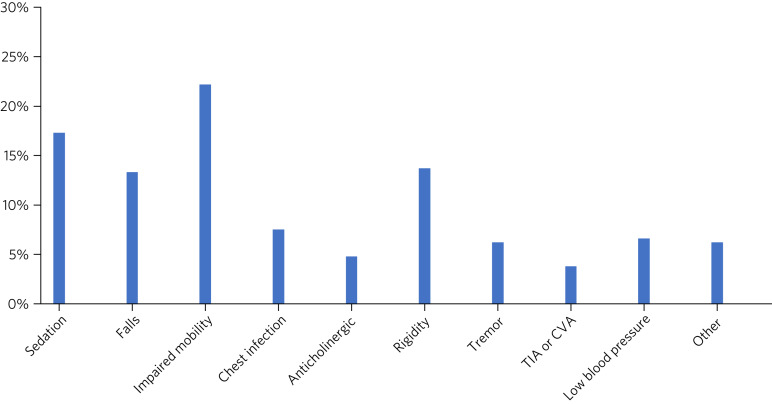
Documented side-effects: proportion of patients experiencing the specified side-effects of antipsychotic medication (*n* = 504) (data from review form). TIA, transient ischemic attack; CVA, cerebrovascular accident.

In 42.7% of forms it was reported that no side-effects had been observed with the antipsychotic drug, whereas the remainder indicated that the patient had experienced one or more side-effect. Figure 3 shows that the most commonly reported side-effects were impaired mobility, sedation, rigidity and falls. Nineteen forms (3.8%) indicated that the patient had a TIA or CVA.

The resulting action taken following the antipsychotic review was as follows: in 61.3% of cases there was no change in medication, in 13.3% review resulted in dose increase, in 12.3% dose was reduced, in 10.5% the antipsychotic was stopped and in 2.6% the antipsychotic was changed.

#### Physical health monitoring on review

Figure 2(b) summarises the proportion of patients who had the specified physical health parameter measured on review. Again, the most frequently performed investigations were blood pressure and pulse; ECG was performed in approximately 50% of cases.

As with the initiation forms, a common response in the comments section was that physical health monitoring had been requested of the GP and results were awaited (34 forms; 6.8%). A comment was made about the patient either refusing or being unable to tolerate blood tests or ECG on 26 (5.2%) forms, and 18 forms (3.6%) highlighted the difficulty in accessing ECG/blood tests because the patient was housebound or otherwise unable to travel.

## Discussion

In the context of a UK programme to enhance the quality of prescribing of antipsychotic drugs in people with dementia, relevant clinical audit data were repeatedly collected by POMH-UK. National data revealed areas of relatively good practice, including consideration of alternatives to antipsychotic medication and clear documentation of target symptoms but areas for improvement, such as the frequency and quality of review of long-term antipsychotic medication, were also identified.^9^ In contrast, SLaM's data revealed good practice in terms of review and monitoring of antipsychotic use, although certain aspects of the pre-treatment screening of patients (e.g. identifying trigger factors for BPSD) required improvement. As a result, SLaM introduced electronic antipsychotic monitoring forms embedded into health records to aid and prompt clinicians to undertake all necessary requirements for pre-treatment screening and monitoring of antipsychotics in people with dementia.

This project examined and evaluated the information routinely collected on electronic monitoring forms regarding antipsychotic prescribing in dementia. Overall form completion was thorough and comprehensive. This may be in part due to the design of the forms, in that many sections are obligatory and the respondent is unable to submit the form without completing them. However, forms reviewed did not appear to be ‘just a tick-box exercise’, evidenced by the frequent use of free-text boxes. Comments in these sections often demonstrated the difficulties experienced in managing symptoms and monitoring of medication.

For over 50% of patients, no record was made of non-pharmacological measures being tried before starting an antipsychotic drug. In 3% of cases, it was documented that it was felt that patient was too unwell to withhold an antipsychotic drug while other non-drug measures were tried first, but underlying reasons for the other instances were not clear. There is limited evidence of any clinically meaningful benefit of antipsychotics in BPSD and any benefit is restricted to psychosis, agitation and aggression,^10,11^ with potential harm (CVAs and mortality) often outweighing the benefits.^12^ Antipsychotics should only be considered in patients with dementia at risk of harming themselves or others or who are experiencing symptoms that are causing them severe distress.^13^ Following this guidance could help to reduce the overall prescribing of antipsychotics in this group. Often staff are not adequately trained to manage these symptoms without the use of drugs. A solution would be to use psychologists and occupational therapists^14,15^ to train and supervise community and in-patient mental health staff in delivering these interventions.

Recorded physical health monitoring at baseline and review was variable. Although nearly three-quarters of patients had blood pressure and pulse monitoring recorded, only about half of patients had an ECG and lipid levels recorded and less than a third had a recorded prolactin level. A reason for not carrying out an ECG was lack of accessibility in patients who were housebound. This highlights the need for more portable ECGs in the trust, so that all patients can access them when needed. In addition, although NICE guidance for the management of psychosis and schizophrenia includes specific recommendations for physical health monitoring when taking antipsychotics, there are no such recommendations in the NICE guidance for dementia. This could explain the low rate of certain monitoring parameters.

On inspection of the review forms, 28.0% indicated that there was no change in symptoms following antipsychotic treatment and about 6.3% indicated that symptoms became worse (34.3% in total). In theory, over a third of the reviews should therefore have led to discontinuation of the antipsychotic drug or a dose increase (if appropriate), but we found that only 13.3% resulted in dose increases, 10.5% in withdrawal and 2.6% in change of antipsychotic drug (26.4% in total). Thus, the antipsychotic should have been stopped in at least an additional 8% of the evaluated forms. Dissemination of these results should encourage clinicians to withdraw antipsychotics where no therapeutic benefits are seen, thereby reducing the overall rate of inappropriate prescribing of antipsychotics in dementia, especially given the continuing risks of long-term use of these agents.

### Strengths and limitations

A strength of this project was that we were able to collect data from all forms completed over 2 years rather than taking a smaller, random sample. A limitation is that an earlier study^5^ carried out by the department estimated that 40% of patients who were prescribed antipsychotics for BPSD did not have a completed form, so we were unable to capture and assess the monitoring of antipsychotic medication in these people. A further limitation is that information was gathered exclusively from the forms for this study, and we did not attempt a full case-note review for clarification. This may be particularly significant for the physical health monitoring, where it was common that the clinician indicated that all the investigations had been performed but did not give further details (e.g. date of the test and the result). Furthermore, if a patient had been on an antipsychotic for a long time, it is likely that they had multiple review forms. It is possible that this could skew the results, for example by overstating the frequency of a particular side-effect.

### Recommendations and areas for improvement

We propose that electronic forms of this nature can facilitate the safe prescribing and monitoring of antipsychotic drugs in dementia. Our current forms capture all the standards required for the POMH-UK audit, as well as additional data on physical health monitoring. We identified areas for improvement, including the need for improved recording of non-pharmacological methods used for BPSD and regarding the routine provision of portable ECGs. In addition, we identified that at least 8% of reviews should have resulted in stopping the antipsychotic owing to lack of therapeutic benefit. We plan to explore ways of adapting the forms so that an alert appears prompting clinicians to stop the antipsychotic when the box for ‘no benefit’ has been ticked, thereby reducing inappropriate prescribing. Similar prompts could be used for other areas requiring improvements, for example the low use of non-pharmacological interventions. We also encourage national guidelines to make recommendations regarding physical health monitoring in patients with dementia taking antipsychotic drugs.

## Data Availability

Data available upon request from corresponding author.

## Supplementary material

For supplementary material accompanying this paper visit http://doi.org/10.1192/bjb.2021.70. click here to view supplementary material

## About the authors

**Helen Anderson** is a higher trainee in dual general and older adult psychiatry with South London and Maudsley NHS Foundation Trust (SLaM), UK. **Anna Kolliakou** is a clinical informatics interface and network lead in the Department of Psychological Medicine, Institute of Psychiatry, Psychology and Neuroscience, King's College London, UK. **Daniel Harwood** is a consultant psychiatrist with SLaM, UK. **Nicola Funnell** is consultant psychiatrist and Clinical Director of Mental Health Services for Older Adults with SLaM, UK. **Robert Stewart** is a consultant liaison old age psychiatrist with SLaM, and Professor of Psychiatric Epidemiology and Clinical Informatics at King's College London, UK. **Delia Bishara** is a consultant pharmacist in mental health for older adults and dementia with SLaM, UK.
