# Supplementary material for: Antipsychotic monitoring in dementia: quality of completion of antipsychotic monitoring forms in an older adult mental health service
Source: BJPsych Bull. 2022 Oct;46(5):271–7. doi: 10.1192/bjb.2021.70 (PMC9768527; doi:10.1192/bjb.2021.70)
Supplement: Supplementary file 1 [file S205646942100070Xsup001.docx]

**Antipsychotic Monitoring in Dementia - Examining the quality of completion of antipsychotic in dementia monitoring forms in SLaM**

**Supplementary Information**

**Initiation of antipsychotic monitoring form**


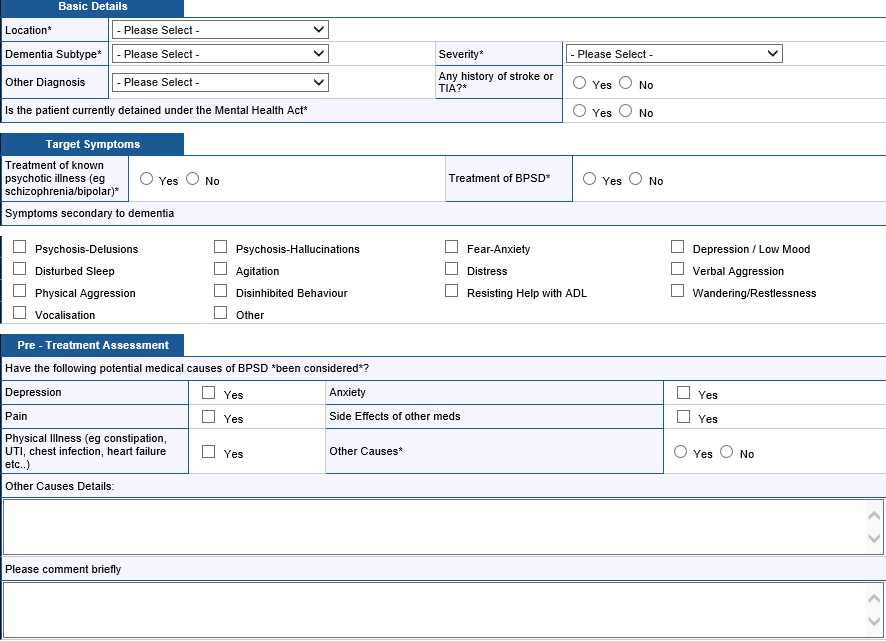

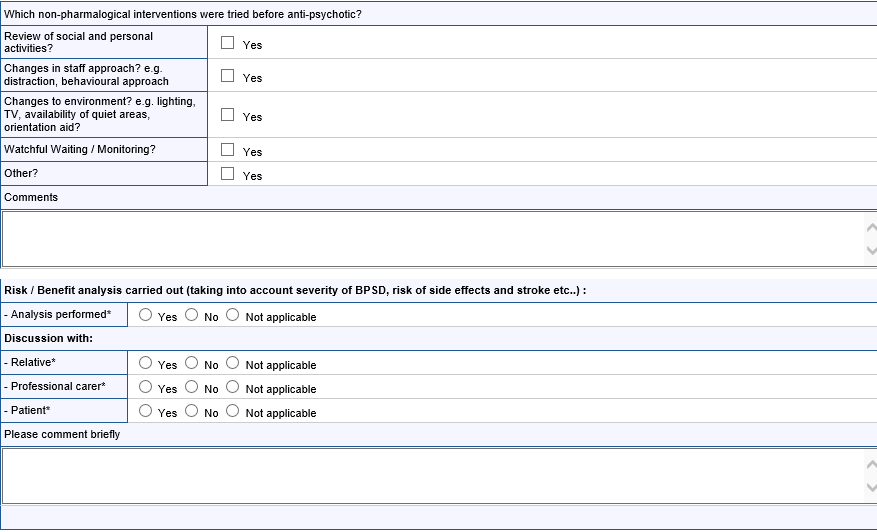


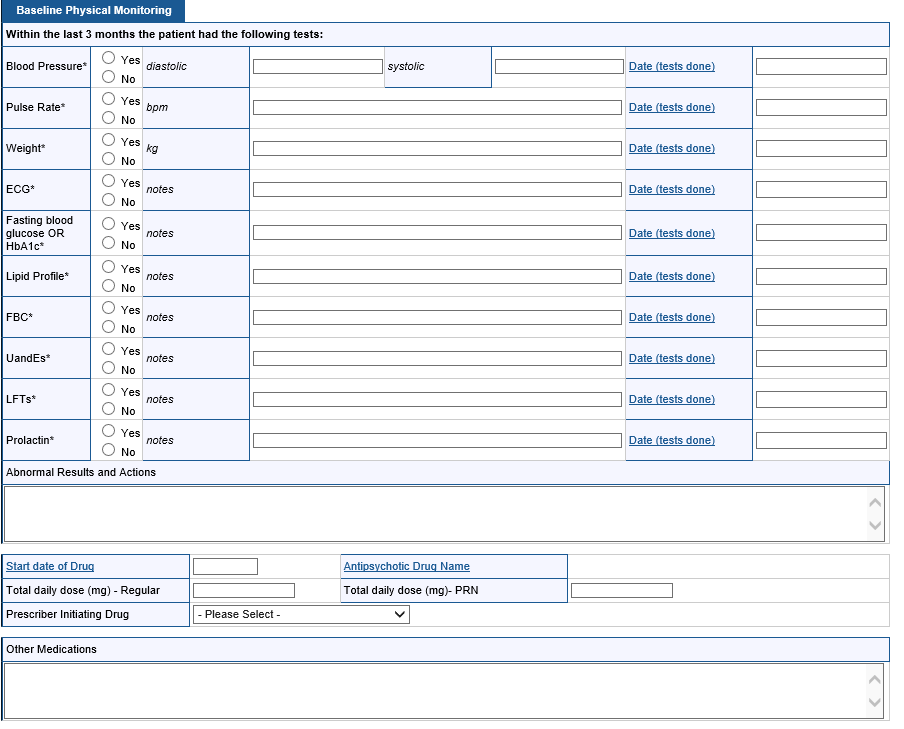


**Review of antipsychotic monitoring form**


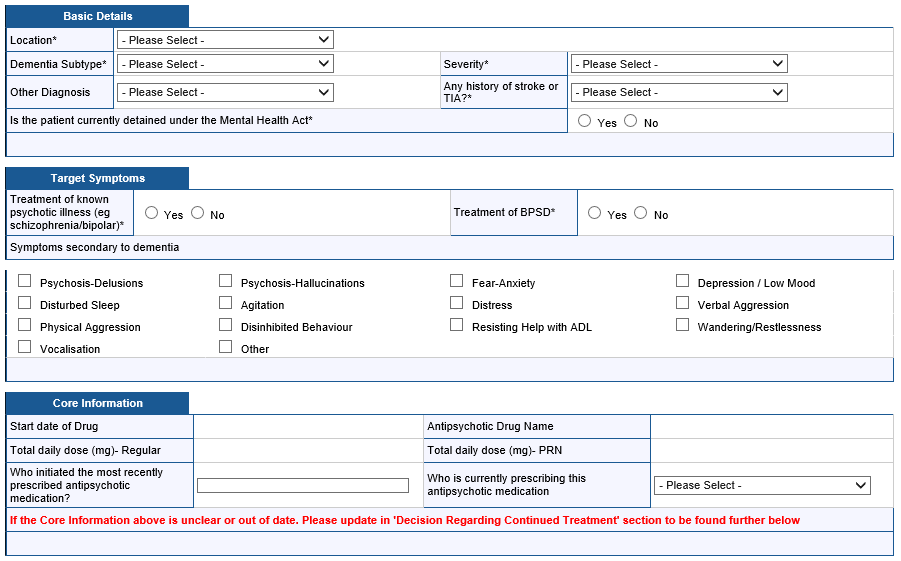


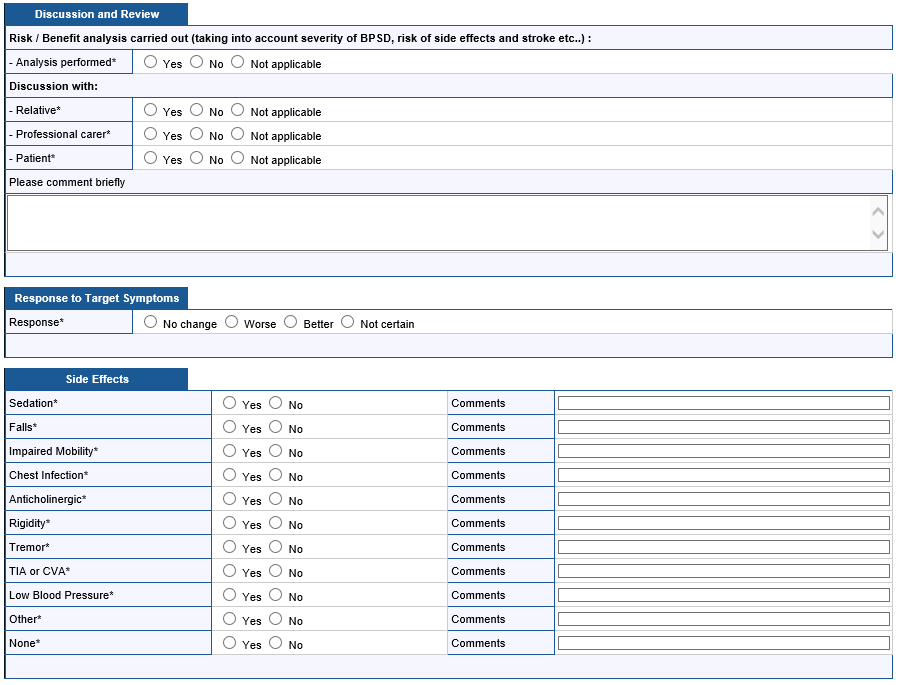


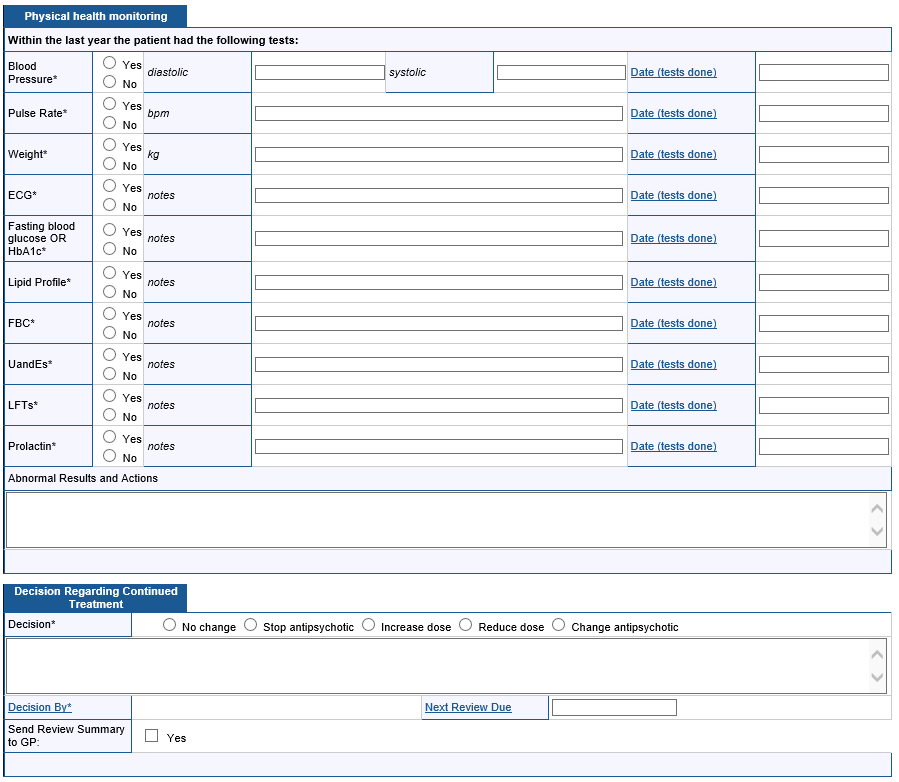


**Supplementary Table 1. Clinical characteristics of the patient group (n = 203)**

| Key clinical characteristics | |  | n (%) |
| --- | --- | --- | --- |
| Dementia subtype |  | |  |
|  | Alzheimer’s dementia | | 108 (53.2) |
|  | ‘Other’ dementia | | 44 (21.7) |
|  | Vascular dementia | | 25 (12.3) |
|  | Mixed dementia | | 11 (5.4) |
|  | Dementia with Lewy bodies | | 9 (4.4) |
|  | Subtype not yet determined | | 5 (2.5) |
|  | Parkinson’s disease dementia | | 1 (0.5) |
| Dementia severity |  | |  |
|  | Mild | | 7 (3.4) |
|  | Moderate | | 97 (47.8) |
|  | Severe | | 99 (48.8) |
| Previous CVA/TIA |  | |  |
|  | Yes | | 33 (16.3) |
|  | No | | 170 (83.7) |
| Detained under the MHA |  | |  |
|  | Yes | | 37 (18.2) |
|  | No | | 166 (81.8) |
